# Imputation of 3 million SNPs in the Arabidopsis regional mapping population

**DOI:** 10.1111/tpj.14659

**Published:** 2020-02-11

**Authors:** Bader Arouisse, Arthur Korte, Fred van Eeuwijk, Willem Kruijer

**Affiliations:** ^1^ Biometris Wageningen University & Research Wageningen Netherlands; ^2^ Centre for Computational and Theoretical Biology University of Würzburg Würzburg Germany

**Keywords:** *Arabidopsis thaliana*, imputation accuracy, regional mapping, 1001 Genomes project, genome‐wide association study

## Abstract

Natural variation has become a prime resource to identify genetic variants that contribute to phenotypic variation. The regional mapping (RegMap) population is one of the most important populations for studying natural variation in *Arabidopsis thaliana*, and has been used in a large number of association studies and in studies on climatic adaptation. However, only 413 RegMap accessions have been completely sequenced, as part of the 1001 Genomes (1001G) Project, while the remaining 894 accessions have only been genotyped with the Affymetrix 250k chip. As a consequence, most association studies involving the RegMap are either restricted to the sequenced accessions, reducing power, or rely on a limited set of SNPs. Here we impute millions of SNPs to the 894 accessions that are exclusive to the RegMap, using the 1135 accessions of the 1001G Project as the reference panel. We assess imputation accuracy using a novel cross‐validation scheme, which we show provides a more reliable measure of accuracy than existing methods. After filtering out low accuracy SNPs, we obtain high‐quality genotypic information for 2029 accessions and 3 million markers. To illustrate the benefits of these imputed data, we reconducted genome‐wide association studies on five stress‐related traits and could identify novel candidate genes.

## Significance Statement

In this study, we imputed the genotypic information of 849 accessions of *Arabidopsis thaliana* with high accuracy. This work makes a large and intensive genotypic resource available for the scientific community and for further meta‐analysis.

## Introduction

*Arabidopsis thaliana* continues to be one of the most important model organisms in plant biology (Somerville and Koornneef, 2002; Koornneef and Meinke, 2010). Its numerous advantages include an easy and manageable growth in controlled conditions, small size, a short generation time, an abundant offspring, and a relatively small nuclear genome.

*Arabidopsis thaliana* occurs as a natural inbred and various genetically distinct varieties, called ecotypes or accessions, have been collected from different natural populations across distinct geographic and environmental ranges (Nordborg *et al.*, 2005; Bevan and Walsh, 2005; Atwell *et al.*, 2010; Cao *et al.*, 2011; Horton *et al.*, 2012; Brennan *et al.*, 2014; Alonso‐Blanco *et al.*, 2016). One of the biggest and most important populations in Arabidopsis is the regional mapping (RegMap) population, containing 1307 accessions that have been genotyped with the Affymetrix Arabidopsis 250K – SNP chip (Horton *et al.*, 2012). The RegMap panel has been used to identify the genetics underlying climate adaptation in *A. thaliana* (Hancock *et al.*, 2011; Lasky *et al.*, 2012; Brachi *et al.*, 2013; Long *et al.*, 2013; Rellstab *et al.*, 2015), and to search for candidate targets of selection using the pairwise haplotype sharing statistic (Toomajian *et al.*, 2006).

Although the average distance between SNPs (~550 bp) in the 250k genotyping data is usually smaller than the average LD decay (~10 kb) (Kim *et al.*, 2007), a large number of unknown variants remains. The 1001 Genomes consortium recently sequenced a population of 1135 accessions (Alonso‐Blanco *et al.*, 2016), and genome‐wide association study (GWAS) on 10 million single nucleotide polymorphisms (SNP) produced associations that could not be found with the markers from the 250K chip. The overlap between the two populations consists of 413 accessions, which means that for 894 of the 1307 RegMap accessions no complete sequence information is available. Imputation of the missing SNPs for these 894 accessions could therefore provide a valuable resource, with a large number of SNPs for 2029 accessions. Our main objective here is to create this resource, and to assess which SNPs can be reliably imputed and used in subsequent analyses. Apart from high accuracy averaged across all accessions, we also aim to achieve good accuracy within the groups of accessions with the minor and major allele. This is particularly relevant when performing subsequent analyses with the imputed data, such as genome‐wide association mapping, where there are often many thousands of markers that just pass a certain minor allele frequency threshold (e.g. 0.05), but whose minor allele count is in the range of 10–30. Biologically, such loci are often highly relevant (Fournier‐Level *et al.*, 2013), but a relatively small number of errors in the imputation can easily lead to decreased power, or false positives.

Imputation methods can be either family or population based, depending on whether haplotypes are inferred from pedigree information or from population‐wide LD patterns. Because pedigree information in *A. thaliana* is mostly missing or unreliable (King *et al.*, 1993), we consider here the methodology implemented in the Beagle software (Browning and Browning, 2016), which is one of the most popular population‐based programs. Although Beagle has been used for plants (Xavier *et al.*, 2016; Pook *et al.*, 2019), its accuracy has hardly been investigated outside humans.

Here we impute all identified SNPs from the 1001G population into the 894 accessions unique to the RegMap panel, and investigate the accuracy of this imputation. We propose a measure of imputation accuracy based on cross‐validation, which we show gives a more reliable predictor of accuracy than the allelic correlation (AR2) used as default parameter in Beagle. After discarding SNPs with too low frequency (minor allele frequency of 0.01) or accuracy, we obtain (depending on the accuracy threshold) a total between 1.4 and 3 million SNPs. To show the benefits of the imputed SNPs, we perform genome‐wide association mapping for five traits from Thoen *et al.*, 2017, obtaining candidate genes for plant growth under several types of biotic and abiotic stress.

## Experimental procedures

### Genotypic data

The 214 051 SNP genotypes of the 1307 RegMap accessions were obtained from the Bergelson laboratory (Horton *et al.*, 2012, see http://bergelson.uchicago.edu/regmap-data/regmap.html/). The genotypic data of the 1001G accessions were obtained from 1001genomes.org (Alonso‐Blanco *et al.*, 2016, see http://1001genomes.org/data/GMI-MPI/releases/v3.1/). The RegMap and 1001G population have 413 accessions in common, while 894 are unique to the RegMap panel.

Both genotypic datasets were subjected to pre‐imputation quality control, consisting of removing SNPs that were present in both datasets, but did not have identical values for all accessions. Additionally, variants only present in the RegMap genotyping were removed, as well as variants with minor allele frequency below 0.01. After quality control there were 3 315 376 SNPs (out of 10.7 M) retained in the 1001G population, and for imputing these for the 894 unique RegMap accessions there were 189 113 SNPs available for all accessions (Table S1). Following the usual terminology from the imputation literature, we refer to the 1001G and unique RegMap accessions with the reference and target set respectively. For imputation on subsets of the data, we indicate the subset between parentheses, e.g. reference (training) for a randomly drawn training set.

### Imputation software

Beagle relies on Bayesian inference for a hidden Markov model, and for each SNP and accession computes the posterior probabilities that the accession has 0, 1, or 2 copies of the reference allele. The imputed value is then the genotype with highest posterior probability. If the highest probability is assigned to one copy of the reference allele (which should not be possible in the inbred populations considered here), we look at which of the two remaining values had the highest probability. However, for around 96% of all imputed values, the maximum posterior probability occurred for the two homozygous possibilities of 0 or 2 copies.

We used Beagle v.5.1, with a window size of 200 kb, an overlap of 12 kb and an effective population size of 250 000. We chose a lower value than the default option (1 million), as effective population size in Arabidopsis has been estimated to range between 250 000 and 300 000 (Cao *et al.*, 2011).

### Imputation accuracy

While the posterior probabilities provide an indication of the uncertainty in a single imputed value, these are not easily translated into an accuracy measure for a given SNP. Such a measure is desirable, since for most purposes one would like to discard SNPs with too many incorrectly imputed accessions. For a given SNP, SNP accuracy is defined as the proportion of correctly imputed accessions (Eqn 1):

(1) $${\text{SNP}}_{\text{accuracy}}=n_{\text{correct}}/n_{\text{total}}.$$

SNP accuracy can, however, vary substantially between allelic groups, and it is desirable to have at least acceptable accuracy for each group. Assuming bi‐allelic SNPs, we therefore define (Eqn 2 and 3):

(2) $$\text{Minor Allele Accuracy}\;=\;n_{\text{correct}}\left(\text{minor}\right)/n_{\text{total}}\left(\text{minor}\right)$$

(3) $$\text{Major Allele Accuracy}=n_{\text{correct}}\left(\text{major}\right)/n_{\text{total}}\left(\text{major}\right)$$

as the SNP accuracy computed over the accessions with, respectively, the minor and major alleles. Because *n*_total_ = *n*_total_(minor) + *n*_total_(major) and *n*_correct_ = *n*_correct_(minor) + *n*_correct_(major), it follows that (Eqn 4):

(4) $${\text{SNP}}_{\text{accuracy}}=\text{(Minor Allele Accuracy}\times \text{MAF)}+\text{(Major Allele Accuracy}\times \left(1-\text{MAF}\right)),$$

where MAF = *n*_total _(minor)/*n*_total_ is the minor allele frequency. Equation (4) shows that for low minor allele frequency (e.g., MAF ≤ 0.05), SNP accuracy is mainly driven by the major allele accuracy. However, in applications such as GWAS the minor allele accuracy is still of importance (Bomba *et al.*, 2017; Yu *et al.*, 2018), as typical MAF threshold (depending on the sample size) are between 0.01 and 0.05, with a minor allele count of at least six. In addition, rare variants are often associated with fitness and provide geographic and climatic local adaptation (Fournier‐Level *et al.*, 2013). For this reason, we will consider here not only the overall SNP accuracy, but also the minor and major allele accuracy.

Additionally, the imputation accuracy per accession was defined as follows (Eqn 5):

(5) $${\text{Accession}}_{\text{Accuracy}}=n{\text{SNP}}_{\text{correct}}/n{\text{SNP}}_{\text{total}},$$

where *n*SNP_correct_ is the number of SNPs imputed correctly and *n*SNP_total_ the total number of SNPs.

The accuracy measures defined above cannot be directly computed, as they depend on the (unknown) true genotypes. We therefore estimate imputation accuracy with Beagle’s AR2 score as well as an empirical approach, based on inner CV. We compare these approaches in a validation study to assess their reliability in terms of minor and major allele accuracy.

### Beagle’s allelic correlation

For each imputed SNP, Beagle computes the allelic correlation (AR2), which is the squared correlation between the allele dosage of the most likely imputed genotype and the allele dosage of the true genotype. It is similar to the SNP accuracy defined in Eqn (1), although the AR2 is scaled differently. The AR2 is estimated from the distribution of imputed posterior genotype probabilities. Browning and Browning (2007) reported that these AR2 estimates are accurate if the provided posterior probability are well calibrated, in the sense that the latter are proportional to the actual probability of incorrect imputation. However, even when the AR2 is high and accurately estimated, the minor allele accuracy may still be low, for example when the MAF is 0.05 and half of the accessions with the minor allele are incorrectly imputed.

### CV estimate of accuracy

As an alternative to the AR2, we estimate accuracy by internal CV on the reference set under consideration. Accessions in the reference set are randomly split into equally large (inner) reference and target sets. SNPs not present in the RegMap are then omitted in the (inner) target set, and imputed (Figure 4). We repeat this 30 times, and for each omitted SNP define CV accuracy as the observed accuracy, averaged over the 30 rounds. This CV estimate can be extended to the minor and major allele accuracies defined in Eqns (2 and 3).

### Validation: comparison of AR2 and CV accuracy

To assess Beagle’s accuracy and to compare the AR2 and CV estimates, we performed 40 random splits, each time dividing the 1001G accessions into a random test set of 227 accessions (20%) and 908 training accessions (80%). SNPs not present in the RegMap are omitted in the test set, and imputed using the training set. We compared the observed accuracy, the AR2 and the CV accuracy. CV accuracy was obtained by performing an inner cross‐validation within the training set (Figure 6). Although eventually no imputation is required for the 1001G accessions, we evaluated the accession accuracy on the same test set, in order to assess the effect of geographic origin. Finally, we used these test sets to assess the calibration of the posterior genotype probabilities.

To evaluate accession accuracy for the 894 unique RegMap accessions, we used a different validation scheme, in which we split the 894 accessions into equally large reference and target panels. Then 20% of the RegMap SNPs were omitted in the target panels to be imputed using the reference panels.

### Genome‐wide association mapping

GWAS on the imputed SNPs was performed using a mixed model implemented in GEMMA (Zhou and Stephens, 2012) (Eqn 6)

(6) $$y=\alpha +X\beta +u+e;u∼N\;\left(0,\;\sigma_{A}^{2}K\right),e∼N\left(0,\;\sigma_{E}^{2}I_{n}\right)$$

where *y* is a *n* × 1 vector of quantitative trait values for *n* accessions, α is the phenotypic mean, *X* is a *n* × 1 vector of marker genotypes, and β is the effect size of the marker. Finally, *u* and *e* are *n* × 1 vectors of random genetic and residual effects, with corresponding variance components σ^2^ *_A_* and σ^2^ *_E_*; *K* is a known *n* × *n* relatedness matrix and *I_n_* is the *n* × *n* identity matrix. In this model all markers are tested individually.

We assess significance using the Bonferroni threshold (−log_10_(0.05/number of tested loci)), as well as a permutation‐based threshold. Following the approach of (Freudenthal *et al.*, 2019), the latter was obtained from GWAS results on 200 random permutations of the phenotype. For each of these we determined the maximum −log10(p) value observed across the genome. The significance threshold was defined as the 95% percentile of these values.

### Haplotype reconstruction and haplotype–trait associations

The partitioning of the genomic regions into segments of strong LD, and the inference of population haplotype patterns from genotype data were performed with the Haploview software (Barrett *et al.*, 2005) and the haplo.stats R‐package (Schaid *et al.*, 2002) using Gabriel *et al.* (2002) algorithm.

Haplotype–trait associations were tested with the haplo.stats package, using an EM‐type algorithm that incorporates both the genotype and the trait, and simultaneously estimates population‐level haplotype frequencies and haplotype–trait associations using an *F*‐test (haplotypes with count below three were discarded).

## Results

### Observed accuracy on the test sets

**Figure 1:**
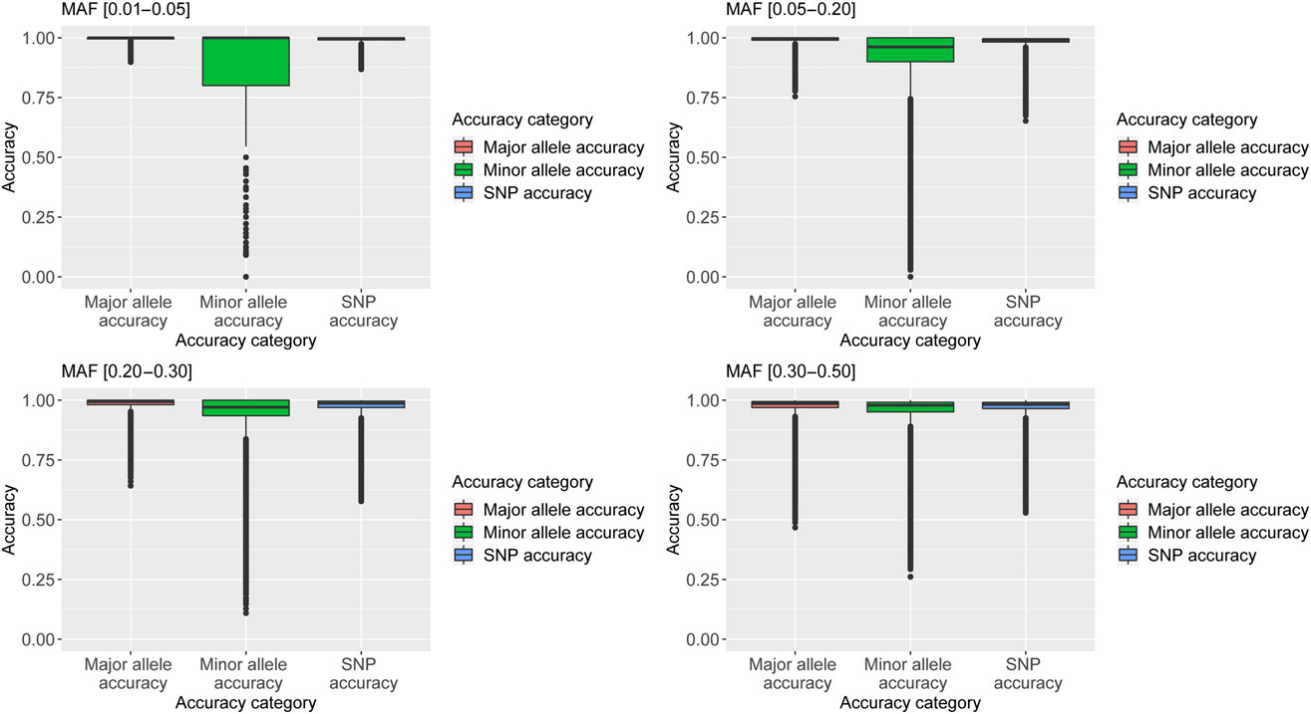
Proportion of correctly imputed single nucleotide polymorphisms (SNPs) (right box) and minor alleles (middle box) and major alleles (left box), in different minor allelic classes.

Beagle performed well on each of our test sets, at least in terms of SNP accuracy averaged over all SNPs (Table S2 and Figure S12). As expected, minor allele accuracy was consistently lower (Figure S11), indicating that errors are more likely to occur for accessions with the minor allele. Accuracy decreased with decreasing allele frequency, and appeared particularly problematic for SNPs with allelic frequency below 0.1, with accuracies below 0.5 for many markers (Figure 1).

**Figure 2:**
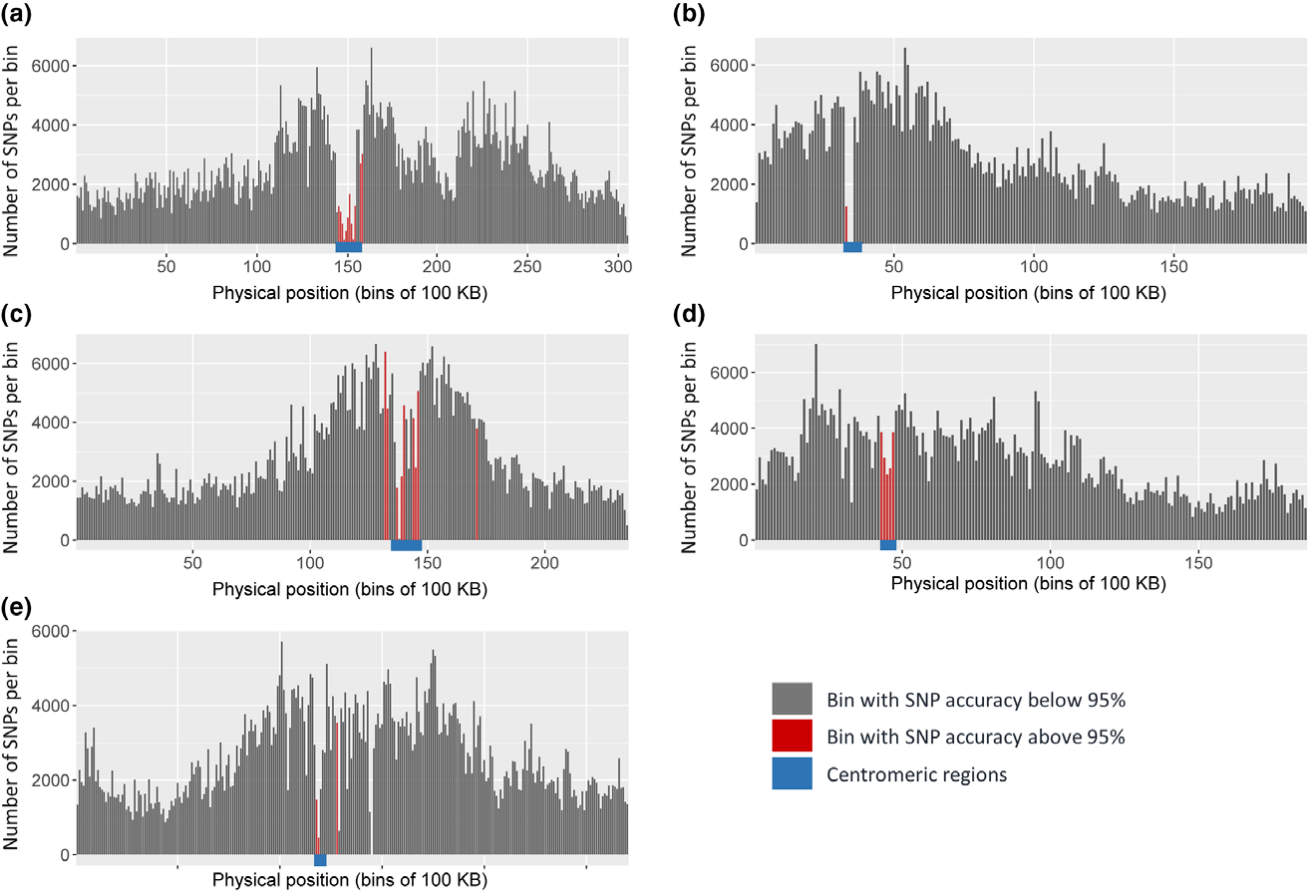
Single nucleotide polymorphism (SNP) density: numbers of imputed SNPs in bins of 100 kb. In the red bins average SNP accuracy is below 0.95.

Figure 2 compares the SNP accuracy and the SNP density over 100 kb bins. Low accuracy bins (in red) occurred mostly in the centromeric regions, which also showed a lower SNP density. Outside the centromeric zones, low accuracy bins mostly occurred in regions with high recombination rates (Horton *et al.*, 2012). Following Browning and Browning (2007), we assessed the calibration on the test sets, by comparing posterior genotype probabilities with the correctness of the corresponding imputations. More than 90% of all sampled accession‐SNP combinations were correctly imputed, with large (>0.9) posterior probabilities on the correct genotype. However, for 10% of the values, the actual correctness (SNP accuracy) was consistently lower than what was suggested by the posterior (Figure S9).

Imputation accuracy per accession ranged between 0.88 and 0.99 for the 1001G accessions and between 0.79 and 0.97 for the unique RegMap accessions (Table S3). Geographically distinct accessions such as Cvi‐0, Etna and Qar (all part of the 1001G population) had comparatively lower imputation accuracy (0.88–0.89), which could be due to the low geographical representation of these accessions, and their genetic distance from the rest of the accessions panel (Figure S14).

### Cross‐validation accuracy and allelic correlation as predictors of accuracy on test sets

**Figure 3:**
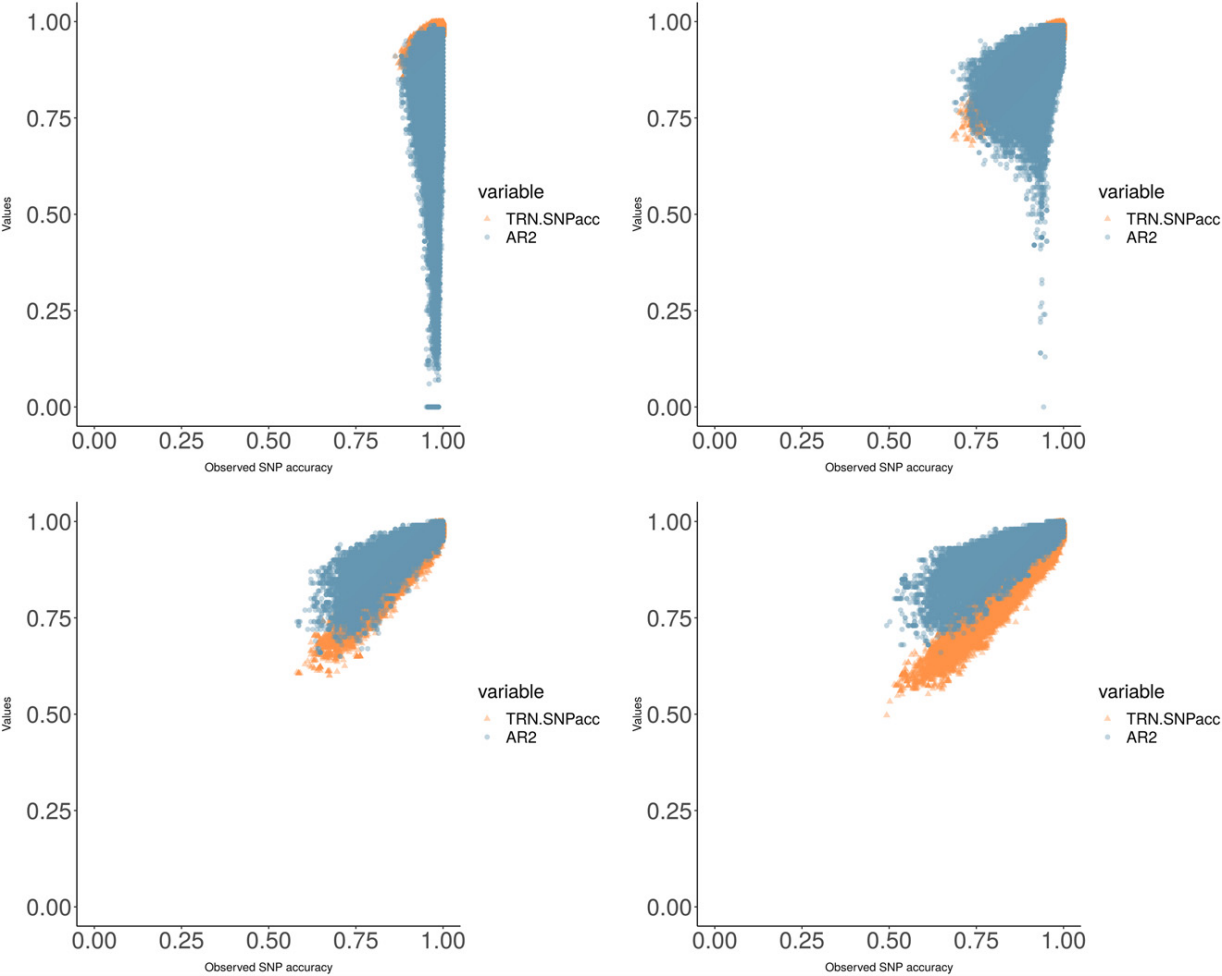
Estimated allelic correlation (AR2) (orange) and on cross‐validation (CV) accuracy (blue) versus the observed accuracy, in different minor allele frequency classes.

Next, we investigated how well the AR2 and cross‐validation (CV) accuracy obtained from the training sets predicted the accuracy observed on the test sets. CV accuracy appeared to be a good predictor of the SNP accuracy as well as the minor and major allele accuracy (Table S4). The correlation with the true accuracies was always larger than 0.75, and in most cases larger than 0.9. Correlation between true accuracies and AR2 values was considerably lower, especially for the major allele accuracy, for SNPs with low MAF (0.19, for SNPs with MAF between 0.01 and 0.05). Although this correlation increased with increasing minor allele frequency, it remained always lower than for the CV accuracy, indicating that the latter is the most reliable predictor of imputation accuracy (Figure 3). While the accuracy itself was generally lower for the minor allele, *estimates* of this accuracy were consistently better for this allele, both for the AR2 and the CV estimates.

### Imputation quality control based on CV accuracy

Using the same test sets, we investigated the effect of quality control based on a fixed threshold for CV accuracy. For thresholds ranging from 0.80 to 0.95, we looked at how many SNPs with low accuracy were still in the data set after quality control, and how many SNPs were left in total (Table 1). For example, with an accuracy threshold of 0.80, we retained 82% of the SNPs with over 97% of them being imputed correctly. For a very stringent accuracy threshold of 0.95, almost all SNPs were accurately imputed (99.9%) and 1.4M SNPs were retained in the data set (42.7%). Although this reduces the number of available markers, it is still about seven times more than the number of SNPs in the 250k data set. Hence the imputation and the use of filters based on CV accuracy resulted in a great improvement in both the quantity and quality of the SNP data.

**Table 1:** Single nucleotide polymorphism (SNP) accuracy, major allele accuracy, and minor allele accuracy of the remaining SNPs, after removing all SNPs with cross‐validation (CV) accuracy below the given threshold

CV‐accuracy threshold	Q‐05^a^	Q‐10^a^	Average	SD	Percentage of accurate SNPs (Number)	Percentage of SNPs left after filtering (Number)
No filter						
Major allele accuracy	0.977	0.983	0.994	0.008	0.93 (2695603)	
Minor allele accuracy	0.583	0.733	0.91	0.168		
SNP accuracy	0.965	0.974	0.99	0.011		
0.8						
Major allele accuracy	0.97	0.981	0.994	0.008	0.9707 (2295485)	0.8204 (2364805)
Minor allele accuracy	0.8	0.852	0.954	0.08		
SNP accuracy	0.96	0.974	0.992	0.01		
0.85						
Major allele accuracy	0.972	0.982	0.995	0.008	0.9775 (2118680)	0.752 (2167394)
Minor allele accuracy	0.826	0.875	0.962	0.069		
SNP accuracy	0.965	0.974	0.992	0.01		
0.9						
Major allele accuracy	0.975	0.983	0.995	0.009	0.9876 (1818613)	0.6388 (1841310)
Minor allele accuracy	0.867	0.909	0.971	0.055		
SNP accuracy	0.969	0.978	0.993	0.01		
0.95						
Major allele accuracy	0.98	0.986	0.996	0.008	0.999 (1230039)	0.4272 (1231243)
Minor allele accuracy	0.923	0.95	0.984	0.036		
SNP accuracy	0.978	0.982	0.994	0.008		

Values are given for one of the 20 test sets (for the other test sets, almost identical results were found). The average, standard deviation (SD) and quantiles were computed over all imputed SNPs with CV accuracy higher than the given threshold. Accurate SNPs are defined by a SNP accuracy (proportion of correctly imputed accessions) above 0.95.

a Q‐5%, 5% quantile; Q‐10%, 10% quantile.

**Figure 4:**
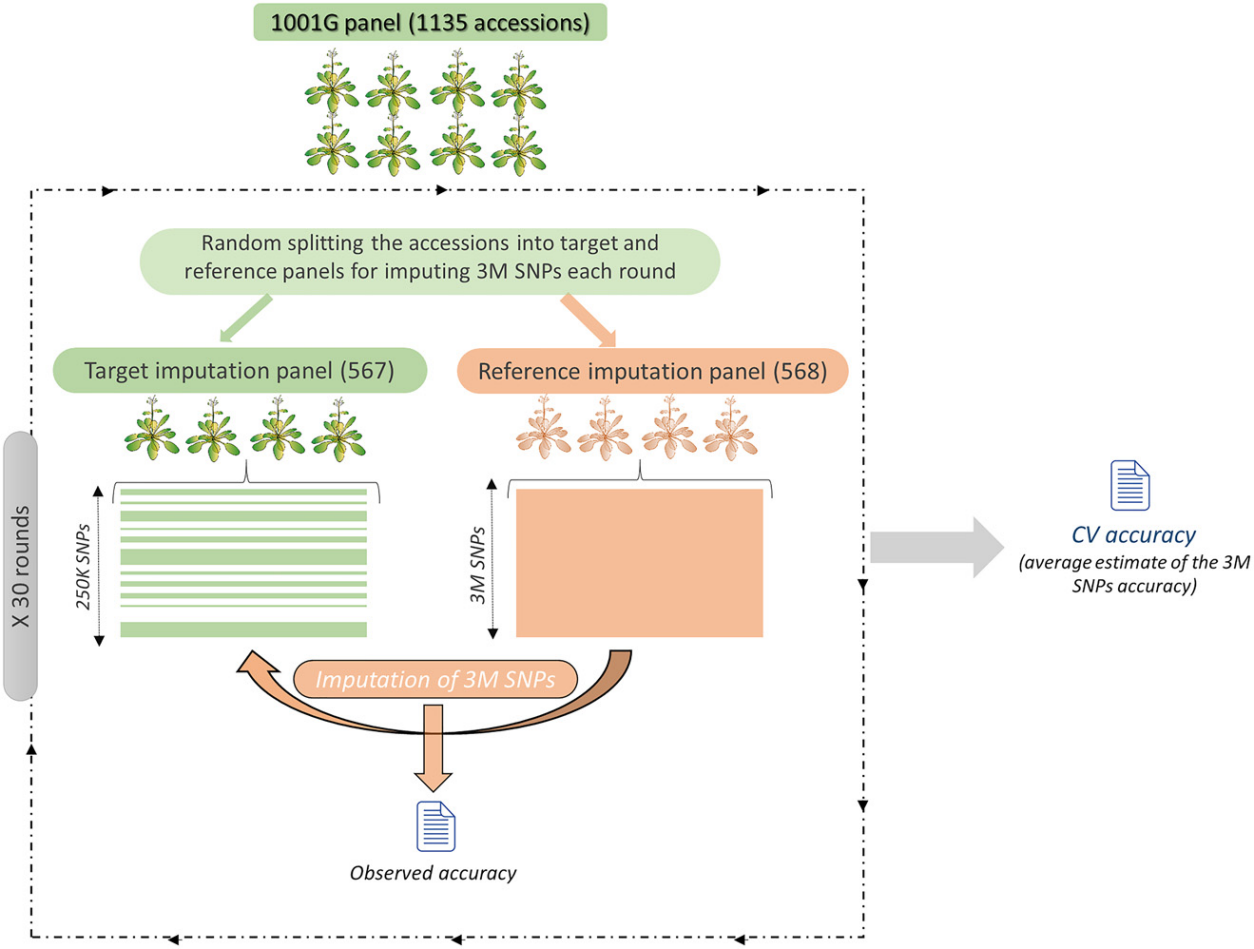
Assessing imputation accuracy with cross‐validation (CV). For a given single nucleotide polymorphism (SNP), the CV accuracy is the observed accuracy (the proportion of correctly imputed accessions), averaged over 30 rounds. In each round, the 1001G population is randomly split into equally large (inner) reference and target sets.

Motivated by these findings on the test sets, we also applied the CV accuracy threshold of 0.95 to our final imputation of all 894 unique RegMap accessions (instead of just 227 test accessions). In this case, CV accuracy was computed using all 1001G accessions (Figure 4), and 1.4m out of the ~3m SNPs had a CV accuracy of at least 0.95.

### Genome‐wide association study

To illustrate the advantage of the imputed SNPs, we performed GWAS on a subset of phenotypes reported by Thoen *et al. *(2017). These data contain measurements on 350 accessions from the *A. thaliana* HapMap population (Figure S1) of biotic and abiotic stress tolerance. The HapMap population is a subset of the RegMap population and contains 149 accessions that are also part of the 1001G panel. GWAS was conducted with the following genotypic data sets: (1) the complete set of ~3m imputed SNPs; (2) the high‐accuracy set of 1.4m, containing SNPs with a CV accuracy of at least 0.95 (3) the 214 051 RegMap SNPs that have been used previously.

**Figure 5:**
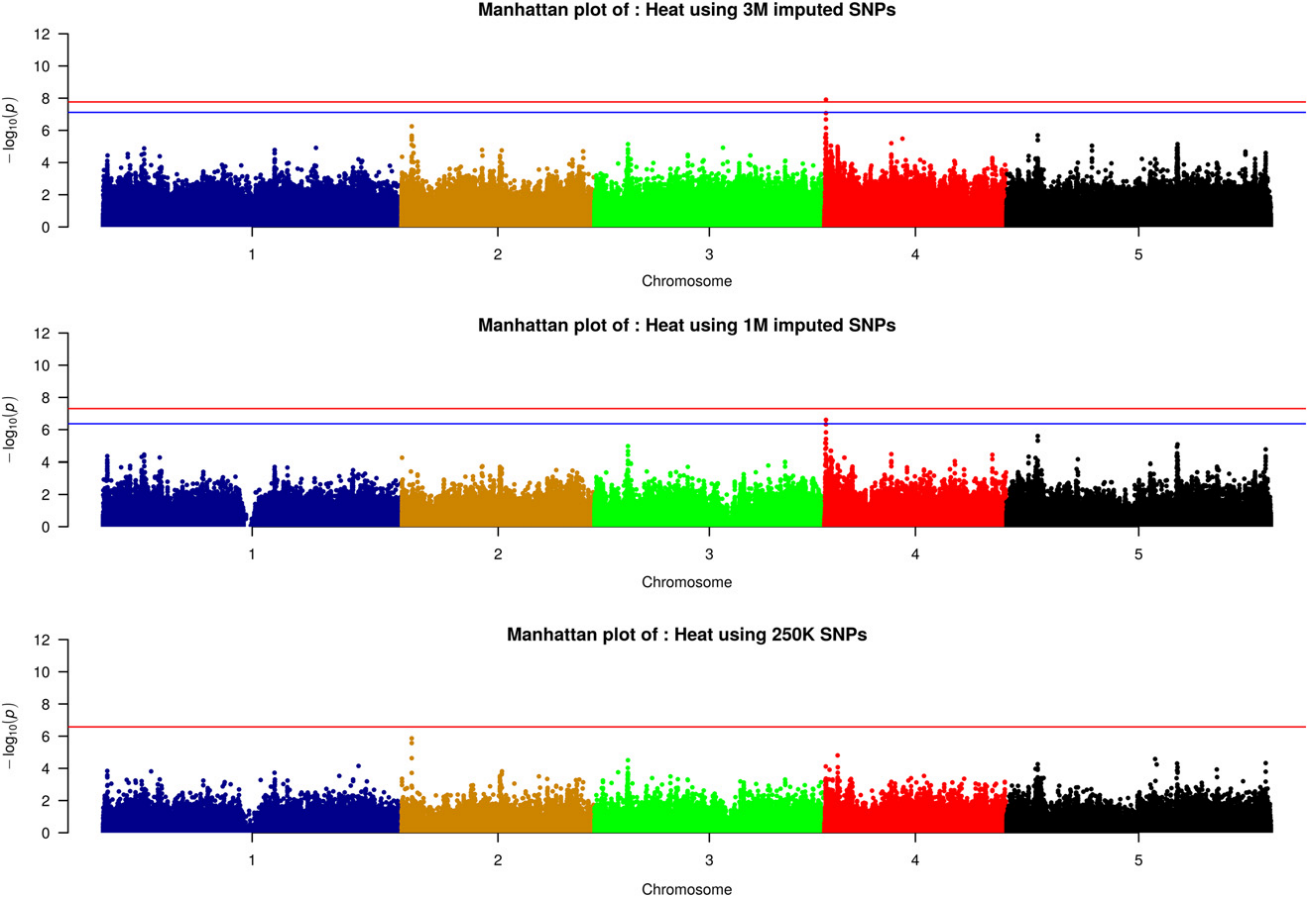
Manhattan plots illustrating the genome‐wide association analysis of growth reduction in plants exposed to heat using 3M imputed single nucleotide polymorphisms (SNPs) (upper plot), filtered 1M SNPs (middle plot) and 250K SNPs (lower plot). The red line is a Bonferroni threshold, while the blue line represents a permutation‐based threshold.

GWAS results for the imputed data revealed several significant associations that were not detected with the 250K SNP data (Figures 5 and [Link], [Link], [Link], [Link]), even given the more stringent multiple‐testing thresholds (due to the larger number of tested variants). For instance, for whitefly stress response (Whitefly_2; Figure S4), we found a very significant association (*P* = 2.00E‐10) on chromosome 2 between base pairs (bp) 7 522 037 and 7 572 663. This region includes several genes involved in RNA methyltransferases activity (e.g. AT4G17610), cellular calcium ion homeostasis (e.g. AT2G17260) and kinase activity that play a central role in signalling during pathogen recognition and the subsequent activation of plant defence mechanisms (e.g. AT2G17320).

**Figure 6:**
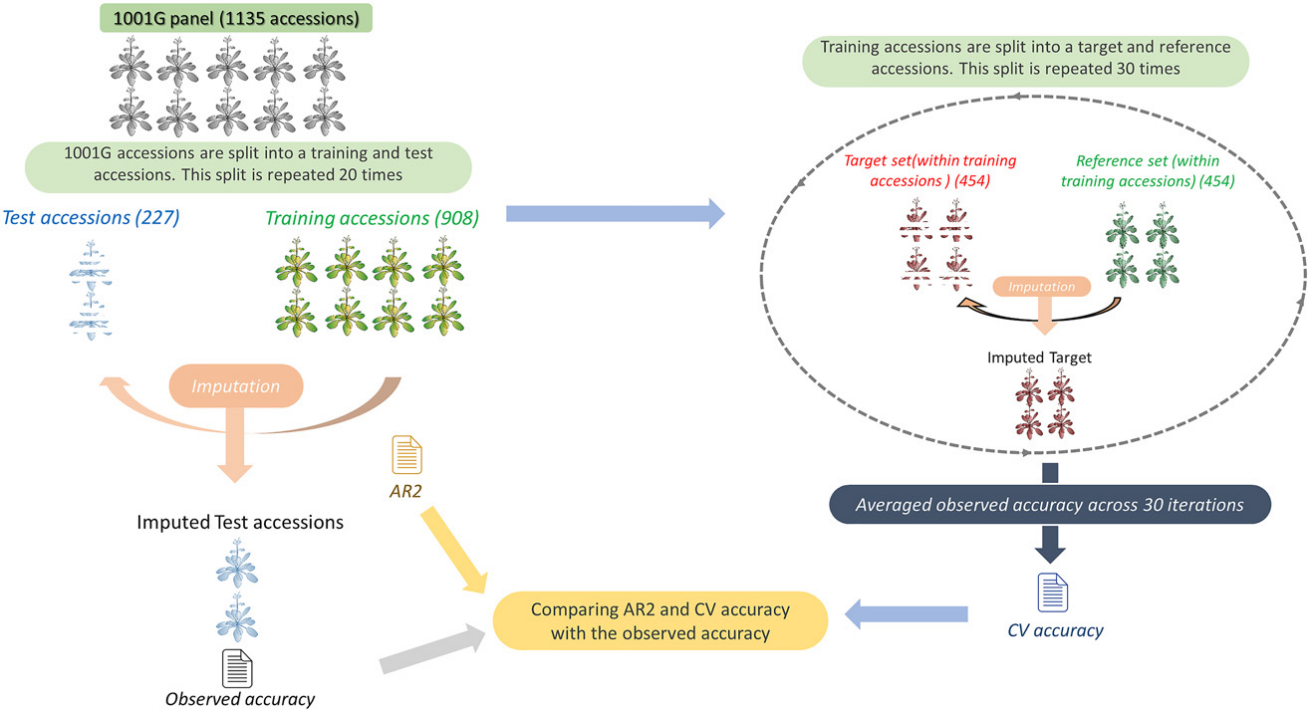
The validation procedure to compare cross‐validation (CV) accuracy and allelic correlation (AR2) as predictors of the single nucleotide polymorphism (SNP) accuracy. First, the 1001G accessions are randomly split into 227 test accessions and 908 training accession, and impute the test accessions. The observed accuracy is then determined by comparing the imputed values and the masked SNP scores in the test set. AR2 values are directly obtained from Beagle, while CV accuracy is based on CV within the training set (right side of the figure). Each time, about 3M SNPs are omitted in the test accessions, leaving only 189K SNPs.

For the response to heat stress (trait ‘Heat’, Figure 5), several novel associations appeared on chromosome 4, which for the 3M SNPs set were significant using both the Bonferroni and the permutation‐based threshold, and almost significant when using the 1.4M SNPs set. The corresponding region (between 104 836 bp and 109 397bp) contains AT4G00240, a gene coding for phospholipase D (PLD) involved in the heat stress response in Arabidopsis seedlings and rice leaves (Mishkind *et al.*, 2009). The novel associations also appeared in GWAS with the original 3M SNPs restricted to 149 accessions belonging to the 1001G population (Figure S7), but without being significant (*P* = 3.64E‐06). This is probably due to the lower power of detecting associated markers in a smaller sample.

No inflation in the GWAS was observed, neither with the 250K data nor with the imputed markers (Figure S8). Consequently, the only costs of using the imputed datasets are the increased computation time and a more stringent multiple‐testing threshold.

### Estimating and testing for haplotype–trait association

We conducted further haplotype analyses on the region containing AT4G00240 (104 836–109 397 bp) (Figure S10), which contains five SNPs from the 250K chip and 52 imputed SNPs, of which three were significant in the GWAS. Haplotype blocks were reconstructed for the five original SNPs from the 250K chip and for the complete set with 57 SNPs, using both the Haploview software and the haplo.stats R‐package (see Experimental procedures). For both SNP sets, Haploview and haplo.stats identified identical blocks.

Using all 57 SNPs, we observed a significant associations for haplotypes Imp_Haplo_29 and Imp_Haplo_30, which appear to give lower heat tolerance (estimated effect of −1.4 for each, and *P*‐value of *F*‐test = 7.84e‐05 (Table S5)). These haplotypes are identical except at position 108 503 bp (marker 52), and they belong to haplotype 250K_Haplo_4 (a haplotype constructed using only SNPs from 250k – SNP chip). For the latter, a less significant haplotype–trait association was found (estimated effect of −0.51801, and *P*‐value of the *F*‐test = 7.035e‐3) (Table S6).

## Discussion

Genotype imputation techniques have a great potential to improve our understanding of the genetic variation that underpins phenotypic diversity. In this study, we imputed 3M SNP genotypes, leading to high‐density genotypic data for 2029 Arabidopsis lines, allowing the community to benefit maximally from existing and future phenotypic data.

Although uncertainty in the imputation can (by using probability scores) be incorporated in subsequent analyses, a much more common strategy is to discard markers whose estimated accuracy is too low. For Beagle, this is usually done by estimating the allelic correlation (AR2), which for the Arabidopsis populations considered here often performed poorly. This may be partially explained by the posterior genotype probabilities, which, in contrast with the results of Browning and Browning (2007) for human populations, were not completely well calibrated. As shown in Browning and Browning (2007), the latter is a requirement for reliable estimation of the true AR2. The suboptimal calibration in our study may be due to a low effective population size, or because Beagle is originally not designed for inbred populations. Another problem with the AR2 is that even if it is high, accuracy may still be low for the accessions with the minor allele.

We therefore proposed a CV approach to assess imputation accuracy, which in our numerical experiments outperformed the AR2. For simplicity our final quality control was based on a threshold for the global SNP accuracy only, but this could be extended with additional thresholds for the major and minor allele accuracy. However, already with the current threshold, the major and minor allele accuracies of the remaining SNPs appear to be high (Table 1), the number of errors being comparable (or lower than) the number of errors occurring in the SNP calling (base call >0.9; Alonso‐Blanco *et al.*, 2016).

Genotype imputation not only increases marker density, but also statistical power for association detection, as datasets from potentially different genotyping technologies and platforms can be combined. In our work, the extended set of SNPs indeed gave more significant associations than an analyses of the subset of 149 accessions for which full sequence data are available or the use of the 250K SNP matrix. By re‐analyzing existing data, we could detect additional genes candidates. Using our data, new and existing phenotypic data could be (re)analyzed, enabling plant scientist to generate new hypothesis about genes involved in traits of their interest.

## Author contributions

BA performed the research. WK, BA, FvE, and AK designed the research. BA and WK wrote the paper, with input from AK and FvE.

## Conflicts of interest

The authors declare that there is no conflict of interest.

## URLs

- The genotypic data of the Regional Mapping was obtained from: http://bergelson.uchicago.edu/regmap-data/regmap.html/.
- The 1001 Genomes Project data set was retrieved from the following link: http://1001genomes.org/data/GMI‐MPI/releases/v3.1/
- https://www.mayo.edu/research/labs/statistical-genetics-genetic-epidemiology/software
- http://zzz.bwh.harvard.edu/plink/
- The newly generated SNP matrix is available here: https://figshare.com/projects/Imputation_of_3_million_SNPs_in_the_Arabidopsis_regional_mapping_population/72887

## Supporting information


**Figure S1.** Accessions composition of 1001 genome and regional mapping project.

**Figure S2.** Imputation workflow of the 3M SNPs to the 894 accessions of the RegMap.

**Figure S3.** Manhattan plots illustrating the genome‐wide association analysis of growth reduction in plants exposed to Whitefly_1 using 3M imputed SNPs (top plot), filtered 1M SNPs (middle plot) and 250K SNPs (bottom plot).). The red line is the Bonferroni threshold while the blue line represents a permutation‐based threshold.

**Figure S4.** Manhattan plots illustrating the genome‐wide association analysis of growth reduction in plants exposed to Whitefly_2 using 3M imputed SNPs (top plot), filtered 1M SNPs (middle plot) and 250K SNPs (bottom plot). The red line is the Bonferroni threshold while the blue line represents a permutation‐based threshold.

**Figure S5.** Manhattan plots illustrating the genome‐wide association analysis of growth reduction in plants exposed to Salt_5 using 3M imputed SNPs (top plot), filtered 1M SNPs (middle plot) and 250K SNPs (bottom plot). The red line is the Bonferroni threshold while the blue line represents a permutation‐based threshold.

**Figure S6**. Manhattan plots illustrating the genome‐wide association analysis of growth reduction in plants exposed to Salt_3 using 3M imputed SNPs (top plot), filtered 1M SNPs (middle plot) and 250K SNPs (bottom plot). The red line is the Bonferroni threshold while the blue line represents a permutation‐based threshold.

**Figure S7.** Manhattan plots illustrating the genome‐wide association analysis of growth reduction in plants exposed to heat using 149 accessions shared between 1001G and the RegMap population, 201 accessions exclusive to the RegMap population, and 350 accessions (the two sets of accessions combined) using 3M, 1M, and 250K SNPs. The red line is the Bonferroni threshold while the blue line represents a permutation‐based threshold.

**Figure S8.** QQ‐plots for GWAS analysis of growth reduction in plants exposed to (a) Heat, (b) Salt_3, (c) Salt_5, (d) Whitefly_1, and (e) Whitefly_2.

**Figure S9**. LD map showing the 16 haplotype blocks encompassing chromosome 4 (104 836–109 397 bp) region, defined by the D’CI method of Gabriel *et al. *(2002).

**Figure S10.** Calibration of posterior genotype probabilities. Imputed genotypes are clustered into bins according to their posterior genotype probabilities. The proportion of imputed genotypes that are imputed correctly are computed for each bin.

**Figure S11.** Boxplot illustrating the major, minor, and SNP imputation accuracy across the 20 test sets.

**Figure S12.** Boxplot showing imputation accuracy for each chromosome across the 20 test sets.

**Figure S13.** Boxplot displays the imputation accuracy using different effective population size (Ne).

**Figure S14.** Multidimensional scaling plot illustrating the imputation accuracy and the genetic distances between the 2029 accessions (894 imputed accessions from RegMap and 1135 reference accessions from 1001G). The latter is based on pairwise identity‐by‐state (IBS) distance computed by PLINK 1.9 (Purcell *et al.*, 2007).

**Table S1**. Overview of SNPs for the 1001G and RegMap populations, with different minor allele frequencies thresholds.
**Table S2**. Imputation accuracy by chromosome, for one of the 20 test sets (for the other test sets, identical results were found; see Figure S12). Averages and standard deviations (SD) were computed over all imputed SNPs on the chromosome in question. Accurate SNPs are defined by a SNP accuracy (proportion of correctly imputed accessions) above 0.95.
**Table S3**. Imputation accuracy per accession.
**Table S4**. Average correlation between CV accuracy (a) and AR2 (b) metrics and the observed SNP accuracy, major allele accuracy, and minor allele accuracy on test sets, by minor allele frequency categories. Minimum and maximum values over 20 test sets are given between parentheses.
**Table S5**. Likelihood‐based haplotype–trait association using imputed and the 250K variant.
**Table S6**. Likelihood‐based haplotype–trait association using only the 250K variants.

## Data Availability

The imputed data and supplementary materials are publicly available in the following figshare repository: https://figshare.com/projects/Imputation_of_3_million_SNPs_in_the_Arabidopsis_regional_mapping_population/72887. The imputed SNPs (with different thresholds for the estimated accuracy) are available under DOI's: https://doi.org/10.6084/m9.figshare.11346833.v1; https://doi.org/10.6084/m9.figshare.11346875.v1; https://doi.org/10.6084/m9.figshare.11346893.v1
