# Supplementary figures and images for: Imputation of 3 million SNPs in the Arabidopsis regional mapping population
Source: Plant J. 2020 Feb 11;102(4):872–82. doi: 10.1111/tpj.14659 (PMC7318218; doi:10.1111/tpj.14659)

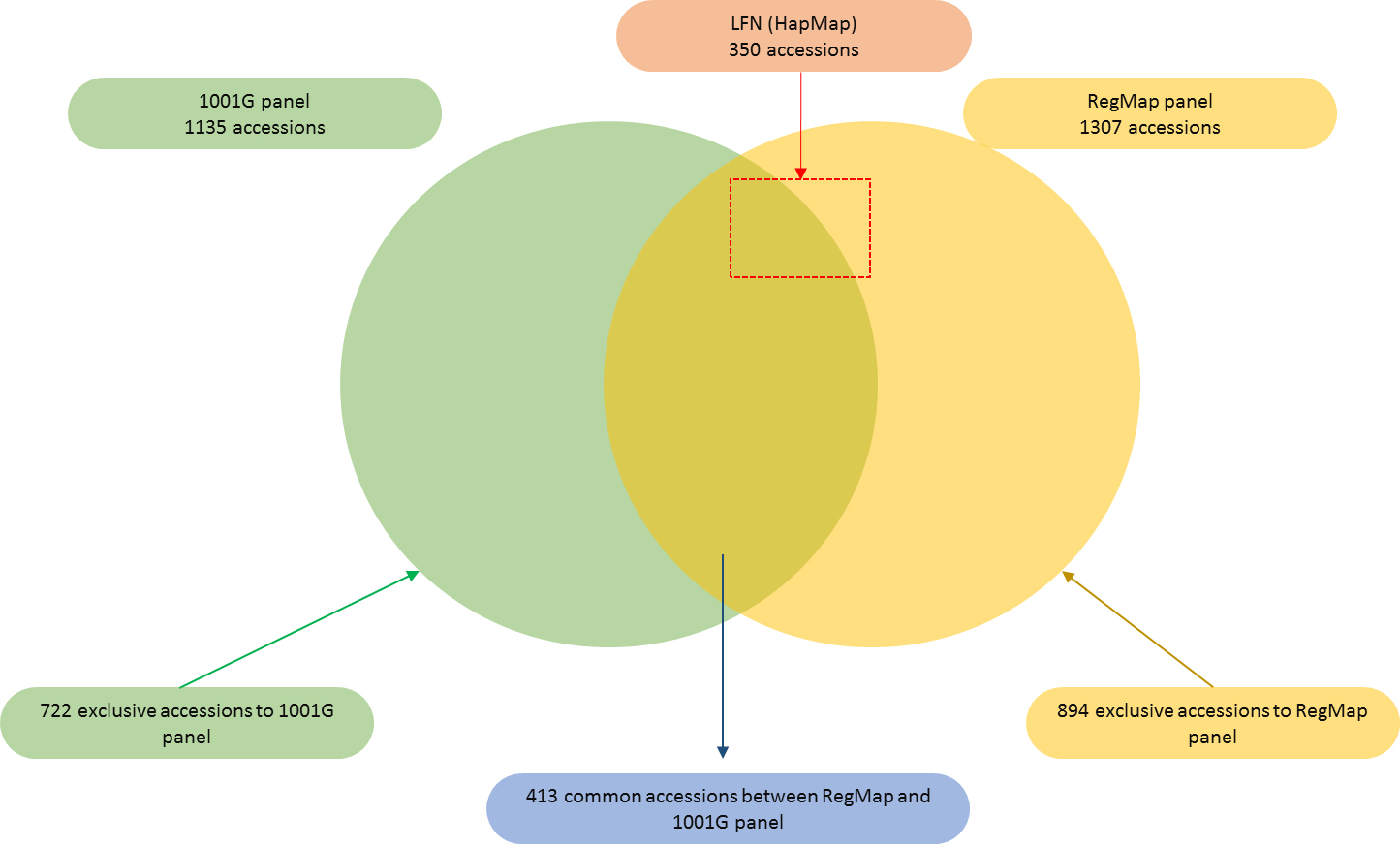

Supplement: Supplementary file 1 — Figure S1. Accessions composition of 1001 genome and regional mapping project. [file TPJ-102-872-s001.jpg]

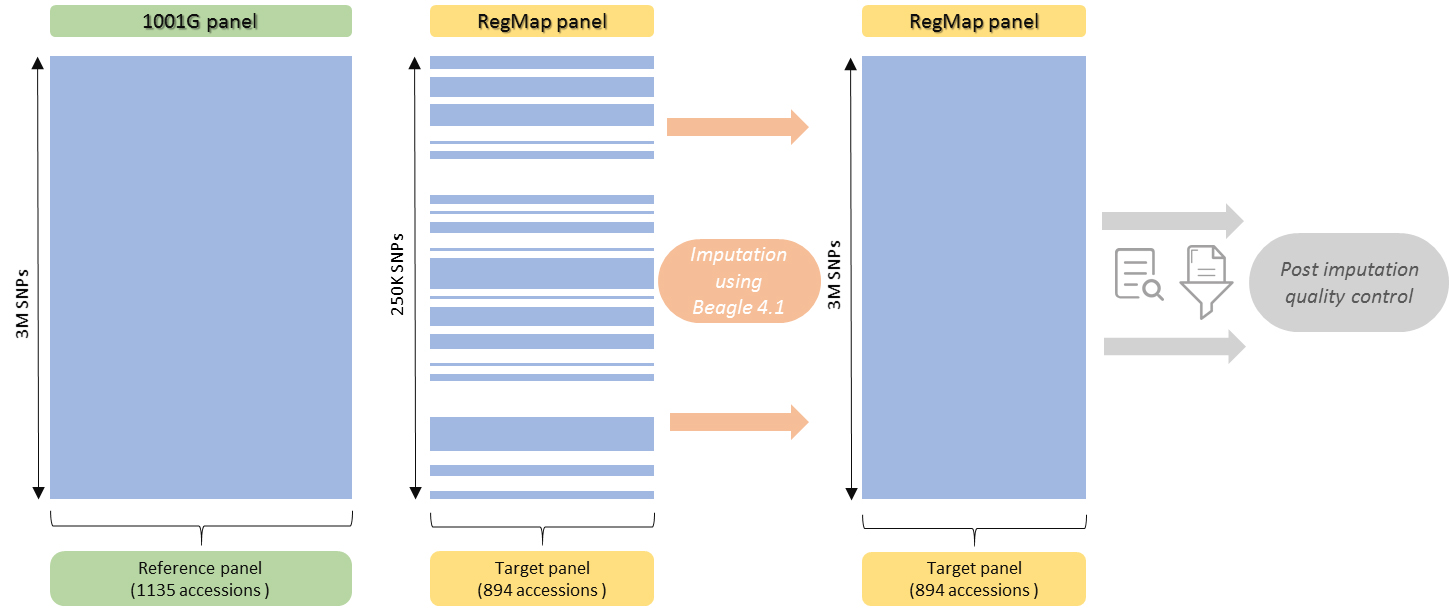

Supplement: Supplementary file 2 — Figure S2. Imputation workflow of the 3M SNPs to the 894 accessions of the RegMap. [file TPJ-102-872-s002.jpg]

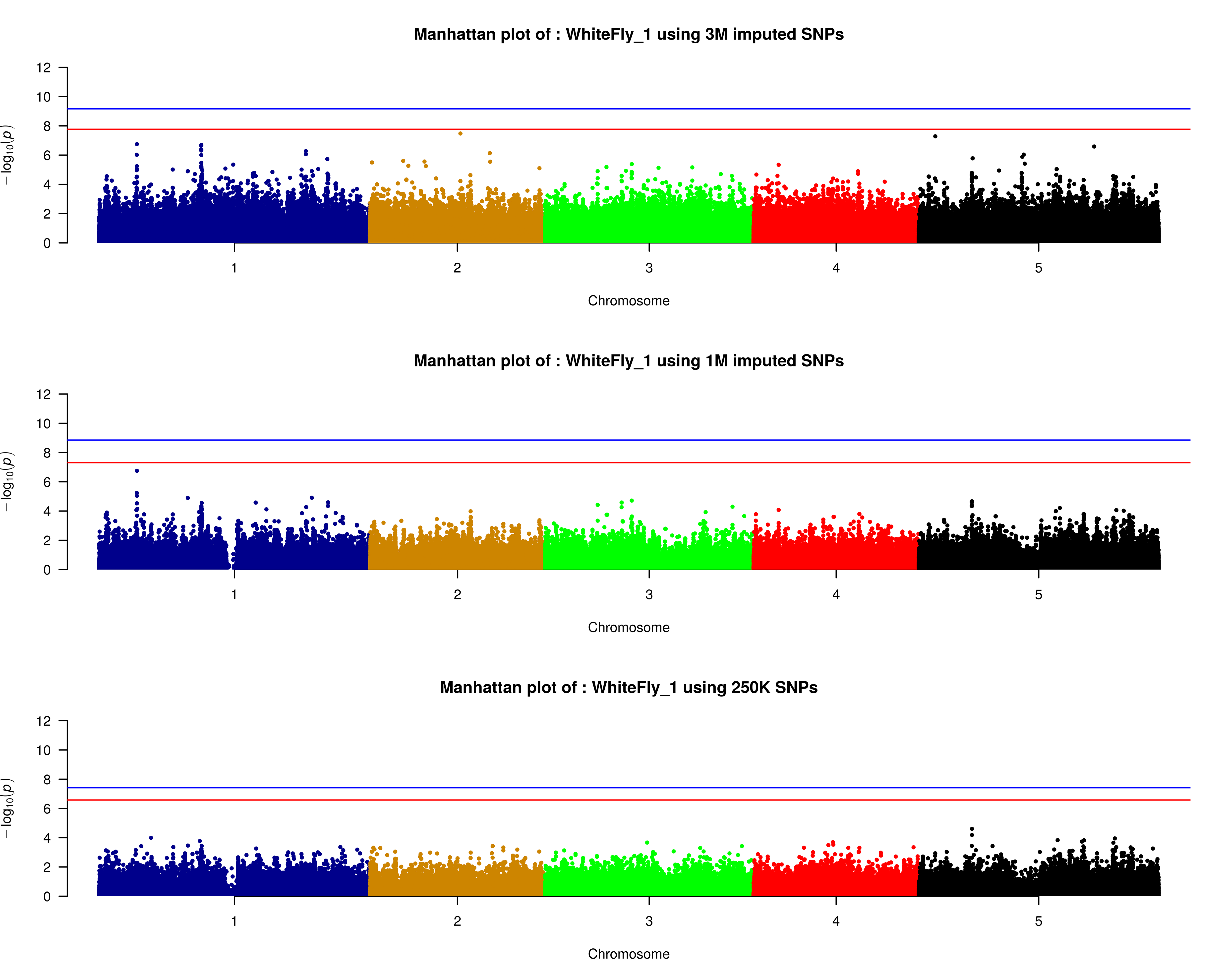

Supplement: Supplementary file 3 — Figure S3. Manhattan plots illustrating the genome‐wide association analysis of growth reduction in plants exposed to Whitefly_1 using 3M imputed SNPs (top plot), filtered 1M SNPs (middle plot) and 250K SNPs (bottom plot).). The red line is the Bonferroni threshold while the blue line represents a permutation‐based threshold. [file TPJ-102-872-s003.png]

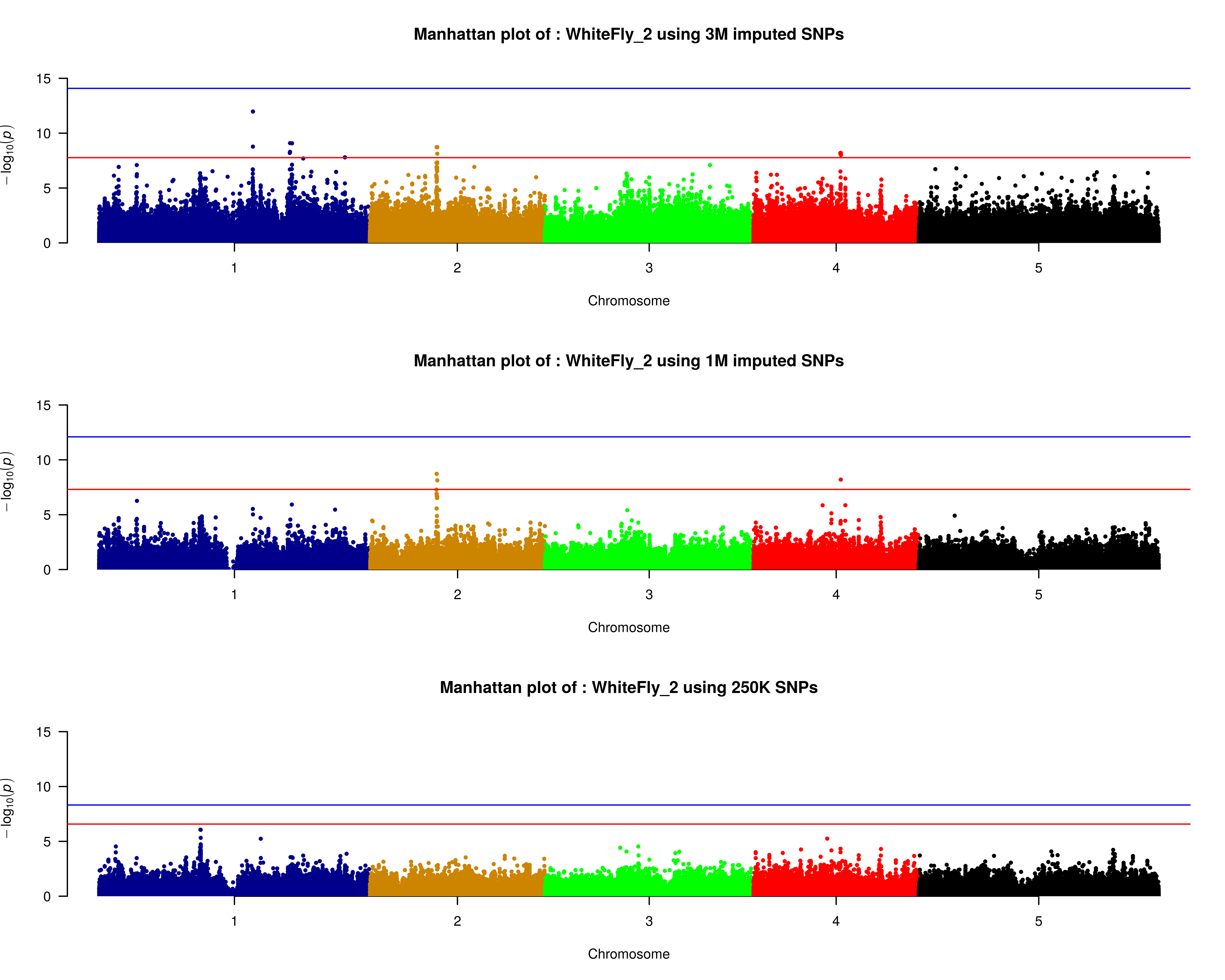

Supplement: Supplementary file 4 — Figure S4. Manhattan plots illustrating the genome‐wide association analysis of growth reduction in plants exposed to Whitefly_2 using 3M imputed SNPs (top plot), filtered 1M SNPs (middle plot) and 250K SNPs (bottom plot). The red line is the Bonferroni threshold while the blue line represents a permutation‐based threshold. [file TPJ-102-872-s004.png]

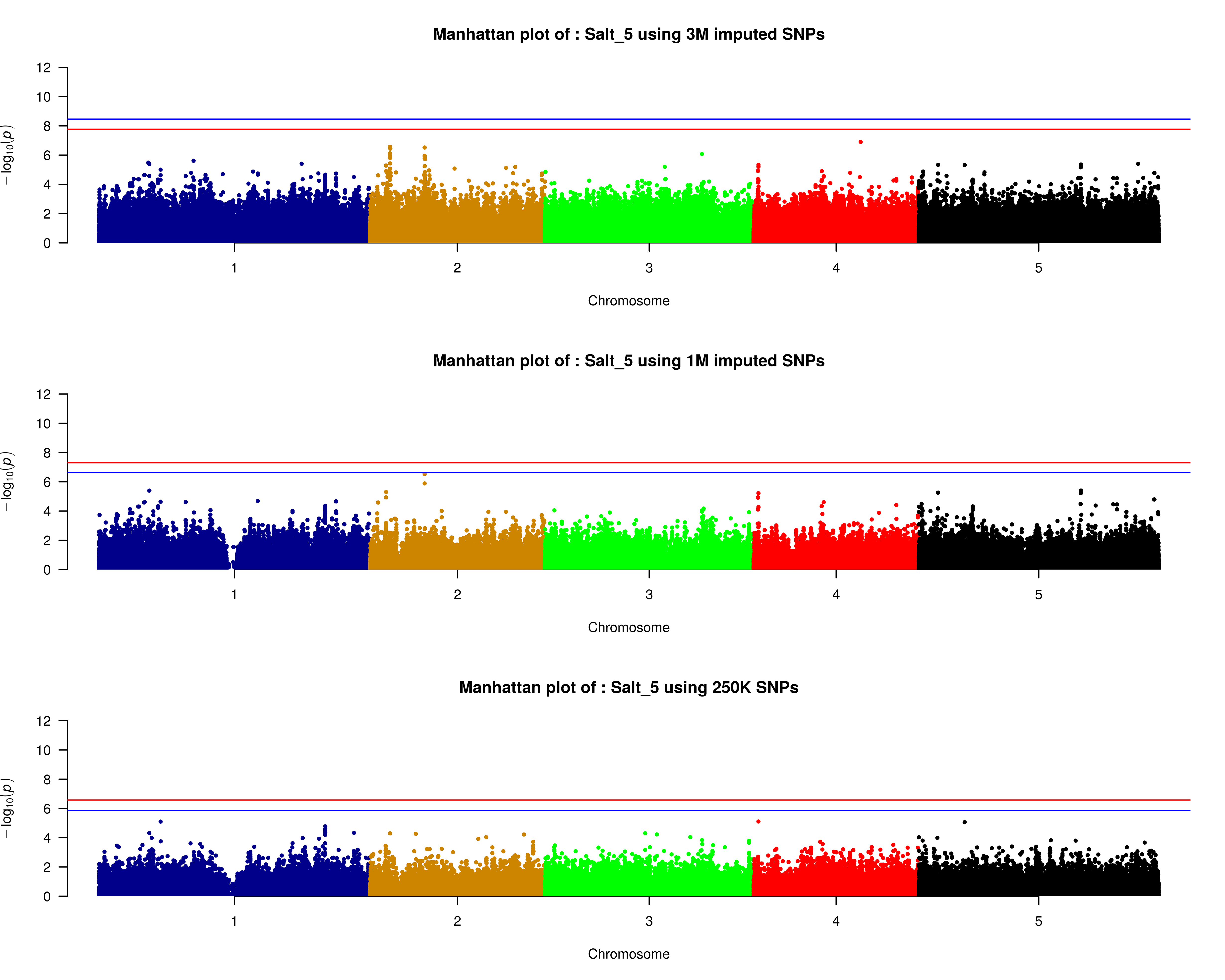

Supplement: Supplementary file 5 — Figure S5. Manhattan plots illustrating the genome‐wide association analysis of growth reduction in plants exposed to Salt_5 using 3M imputed SNPs (top plot), filtered 1M SNPs (middle plot) and 250K SNPs (bottom plot). The red line is the Bonferroni threshold while the blue line represents a permutation‐based threshold. [file TPJ-102-872-s005.png]

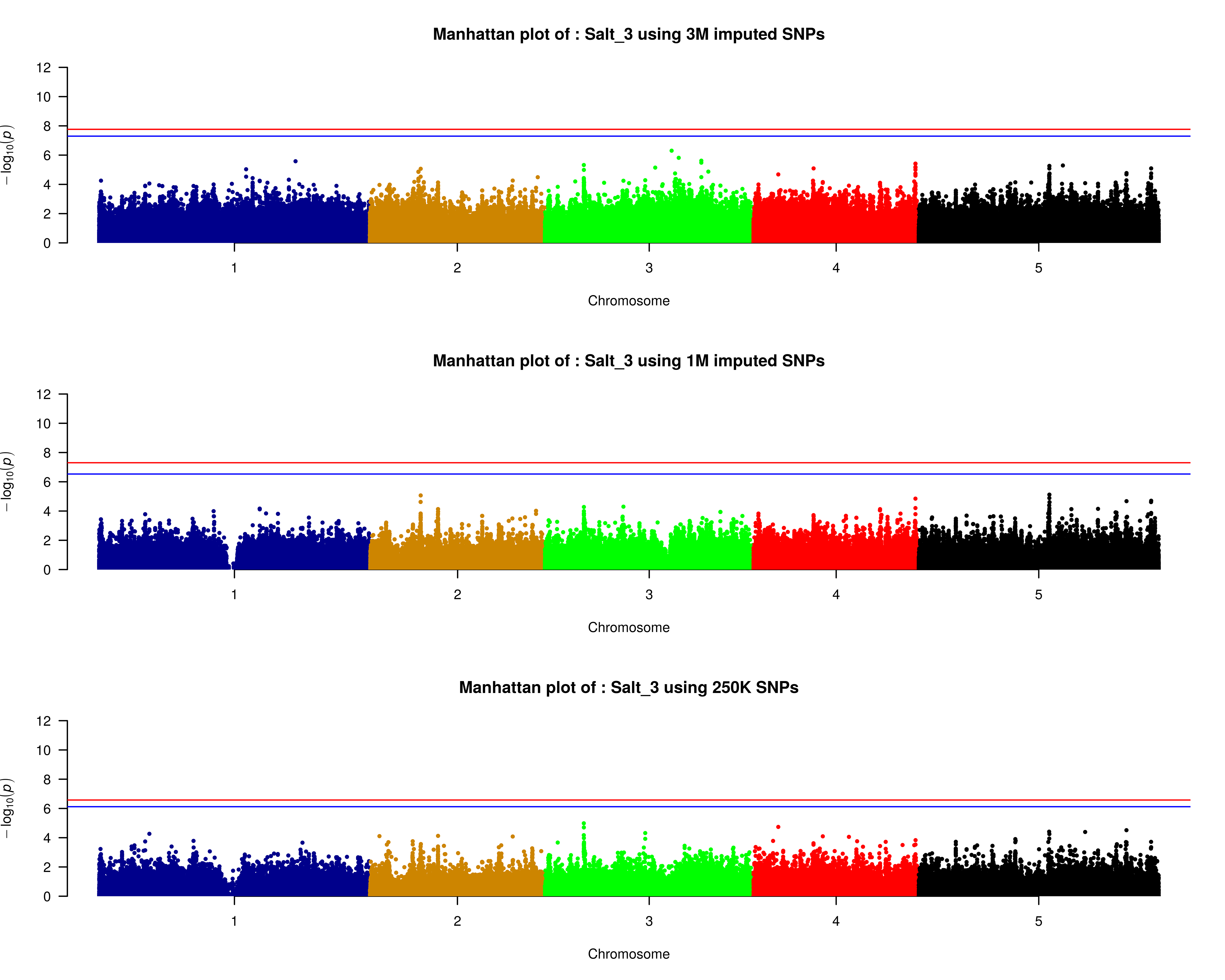

Supplement: Supplementary file 6 — Figure S6. Manhattan plots illustrating the genome‐wide association analysis of growth reduction in plants exposed to Salt_3 using 3M imputed SNPs (top plot), filtered 1M SNPs (middle plot) and 250K SNPs (bottom plot). The red line is the Bonferroni threshold while the blue line represents a permutation‐based threshold. [file TPJ-102-872-s006.png]

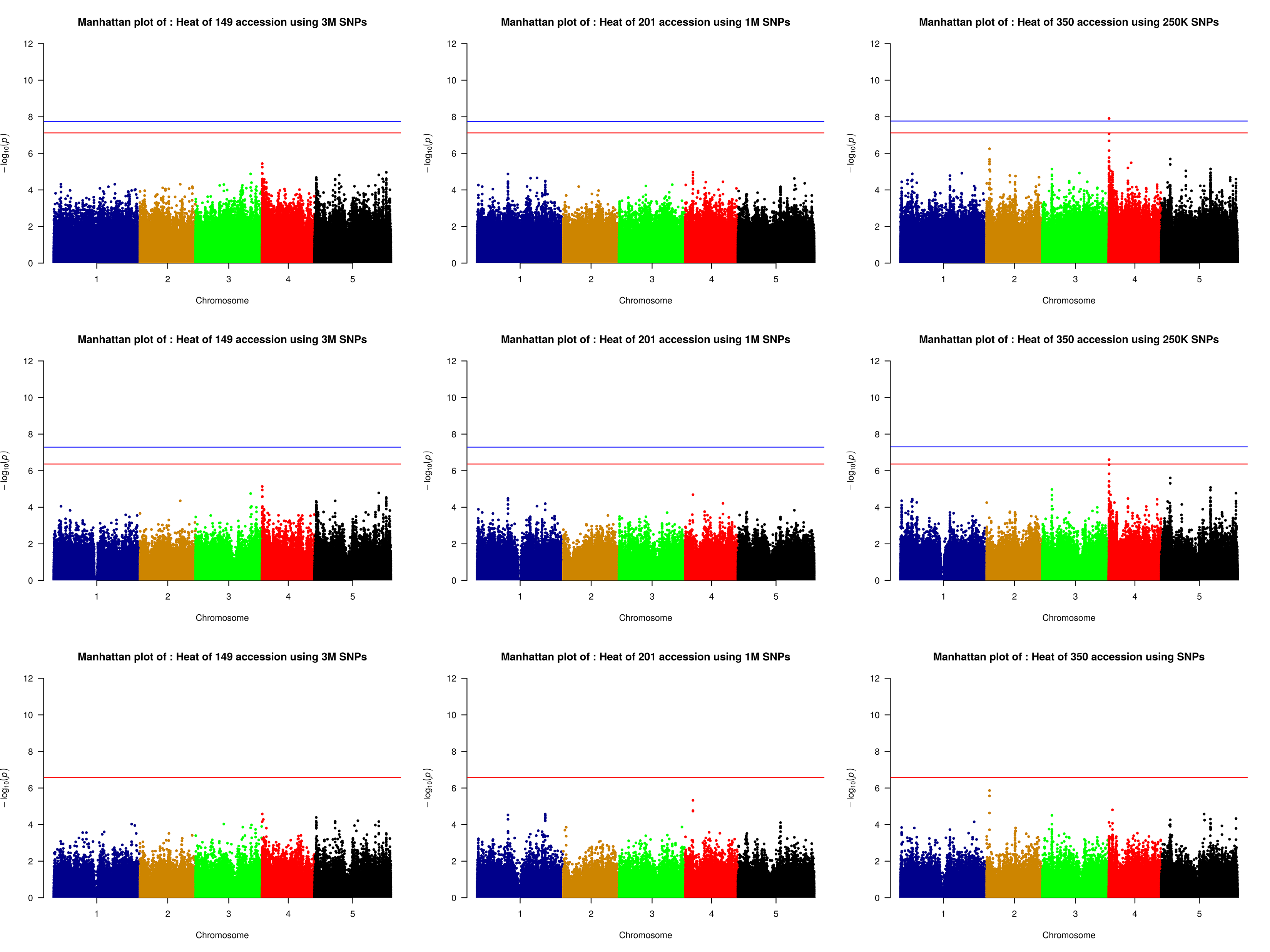

Supplement: Supplementary file 7 — Figure S7. Manhattan plots illustrating the genome‐wide association analysis of growth reduction in plants exposed to heat using 149 accessions shared between 1001G and the RegMap population, 201 accessions exclusive to the RegMap population, and 350 accessions (the two sets of accessions combined) using 3M, 1M, and 250K SNPs. The red line is the Bonferroni threshold while the blue line represents a permutation‐based threshold. [file TPJ-102-872-s007.png]

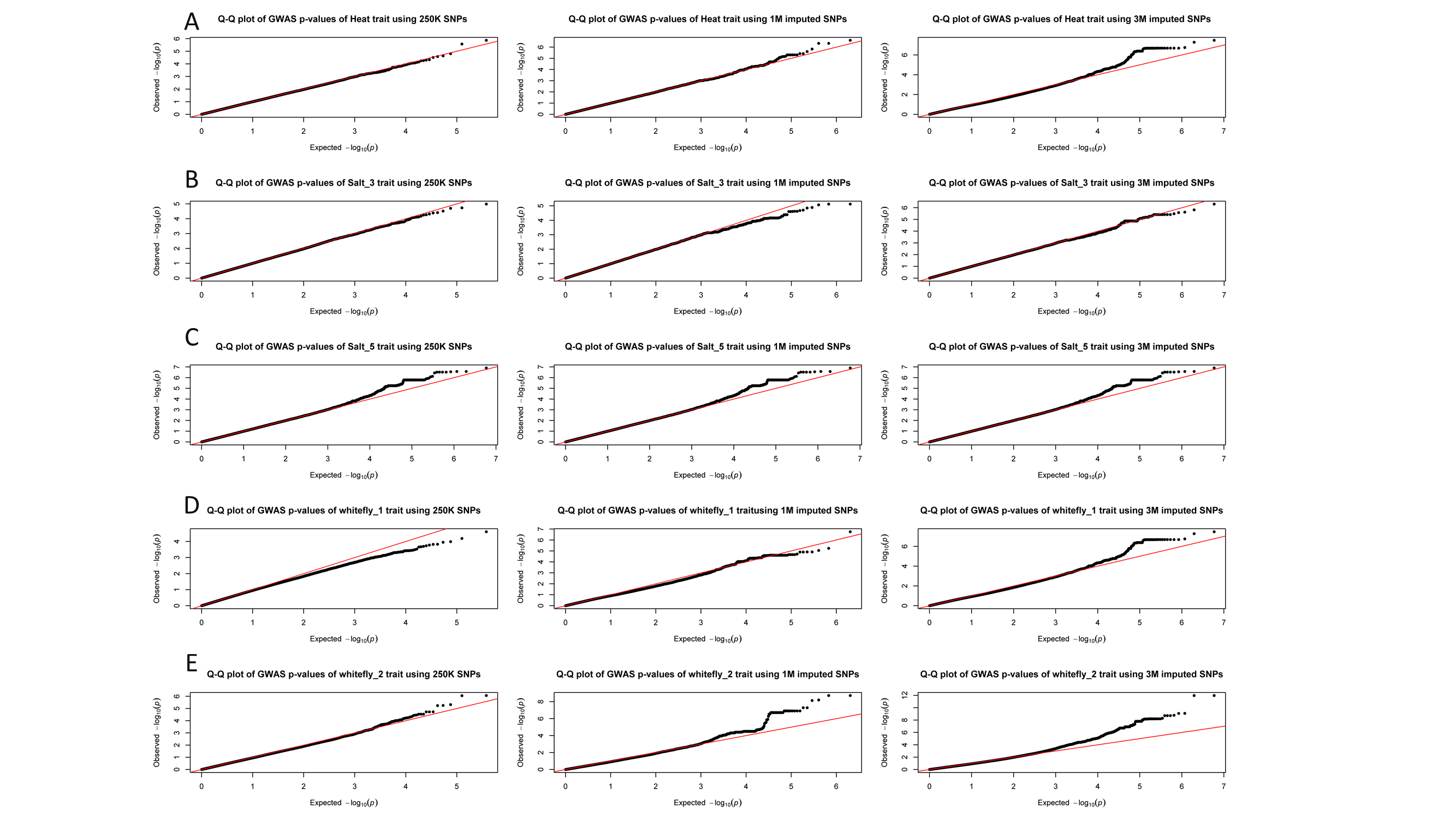

Supplement: Supplementary file 8 — Figure S8. QQ‐plots for GWAS analysis of growth reduction in plants exposed to (a) Heat, (b) Salt_3, (c) Salt_5, (d) Whitefly_1, and (e) Whitefly_2. [file TPJ-102-872-s008.png]

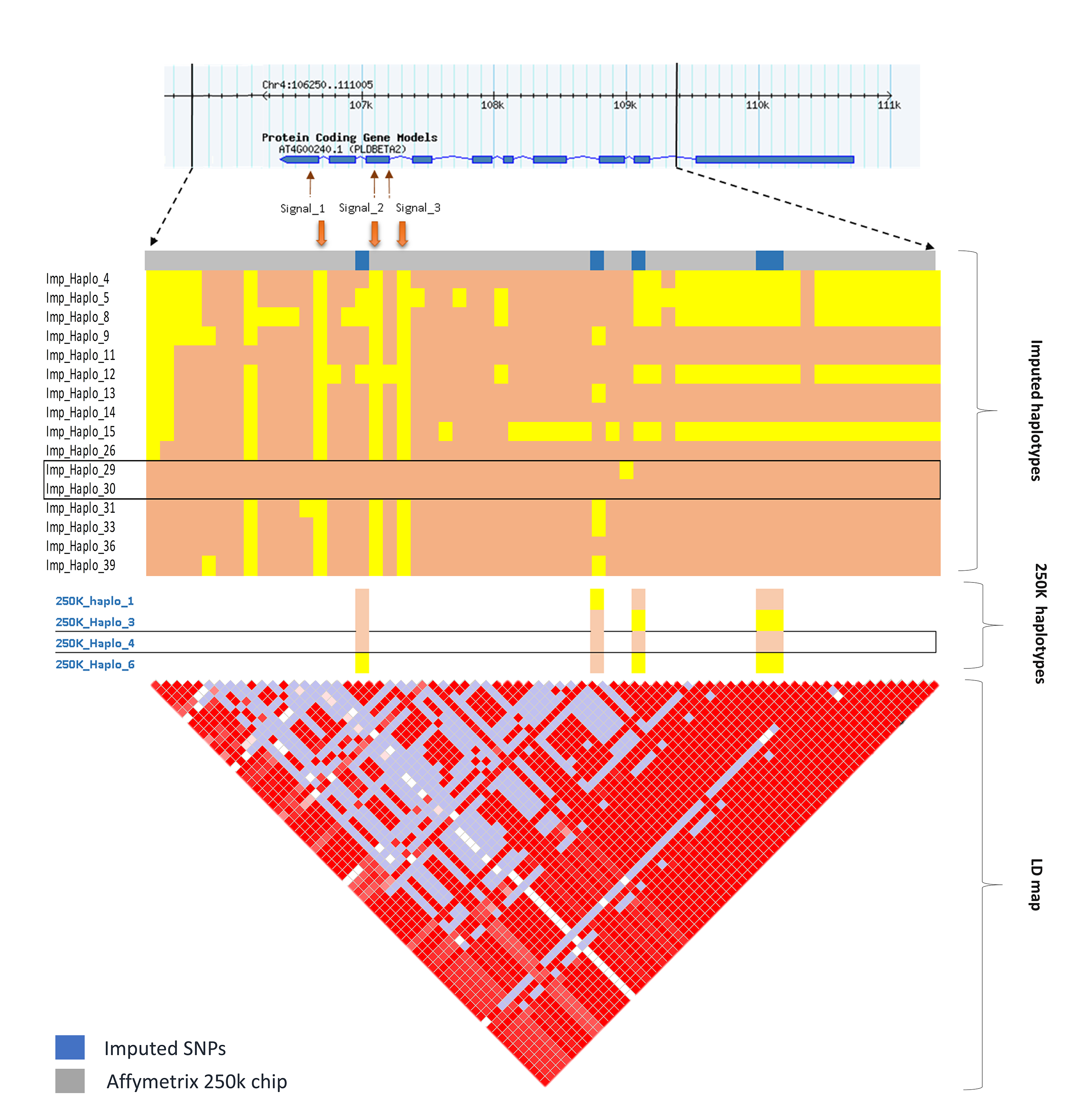

Supplement: Supplementary file 9 — Figure S9. LD map showing the 16 haplotype blocks encompassing chromosome 4 (104 836–109 397 bp) region, defined by the D’CI method of Gabriel et al. (2002). [file TPJ-102-872-s009.png]

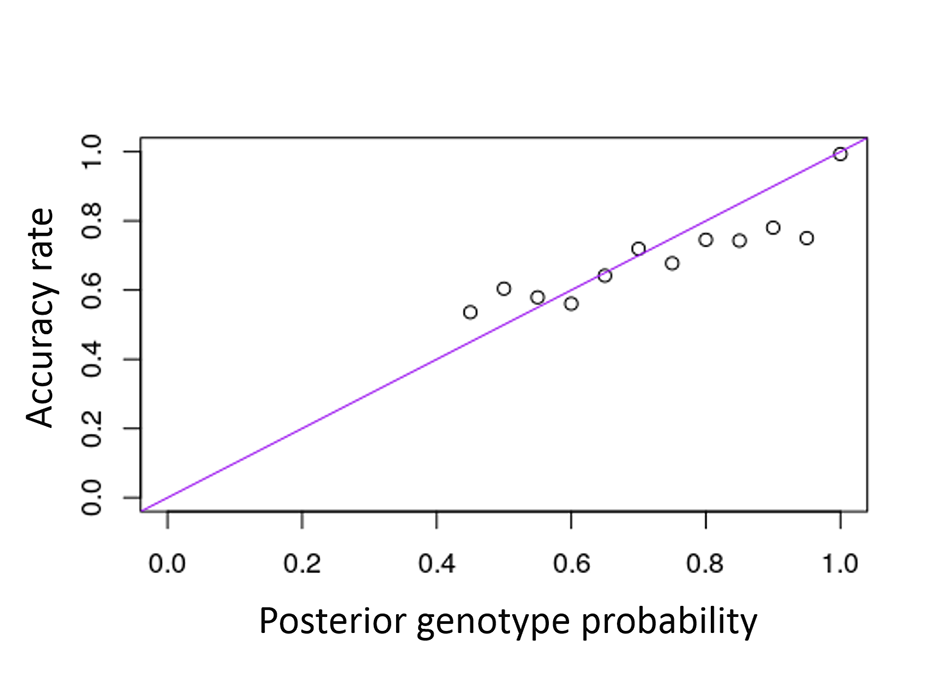

Supplement: Supplementary file 10 — Figure S10. Calibration of posterior genotype probabilities. Imputed genotypes are clustered into bins according to their posterior genotype probabilities. The proportion of imputed genotypes that are imputed correctly are computed for each bin. [file TPJ-102-872-s010.png]

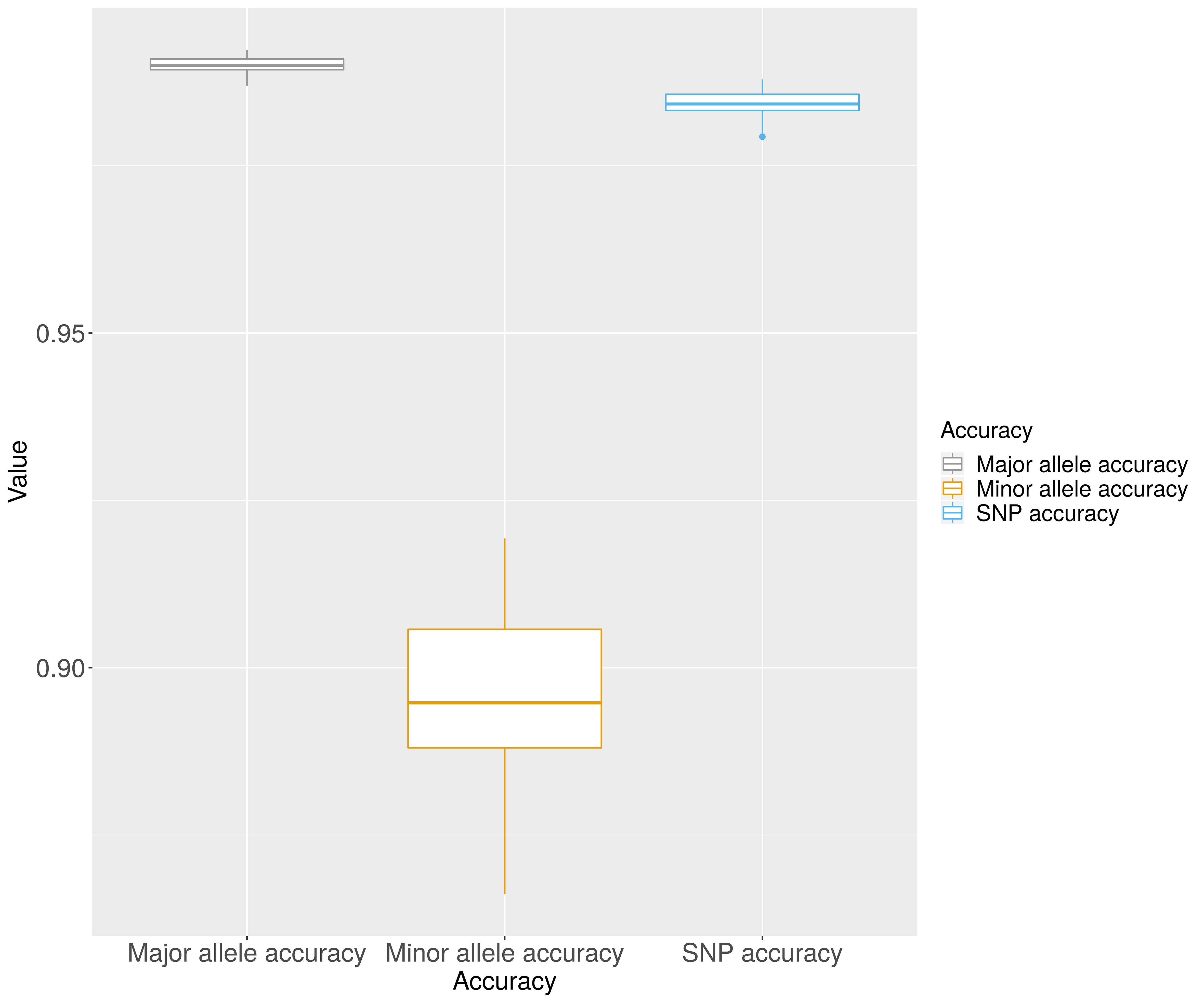

Supplement: Supplementary file 11 — Figure S11. Boxplot illustrating the major, minor, and SNP imputation accuracy across the 20 test sets. [file TPJ-102-872-s011.png]

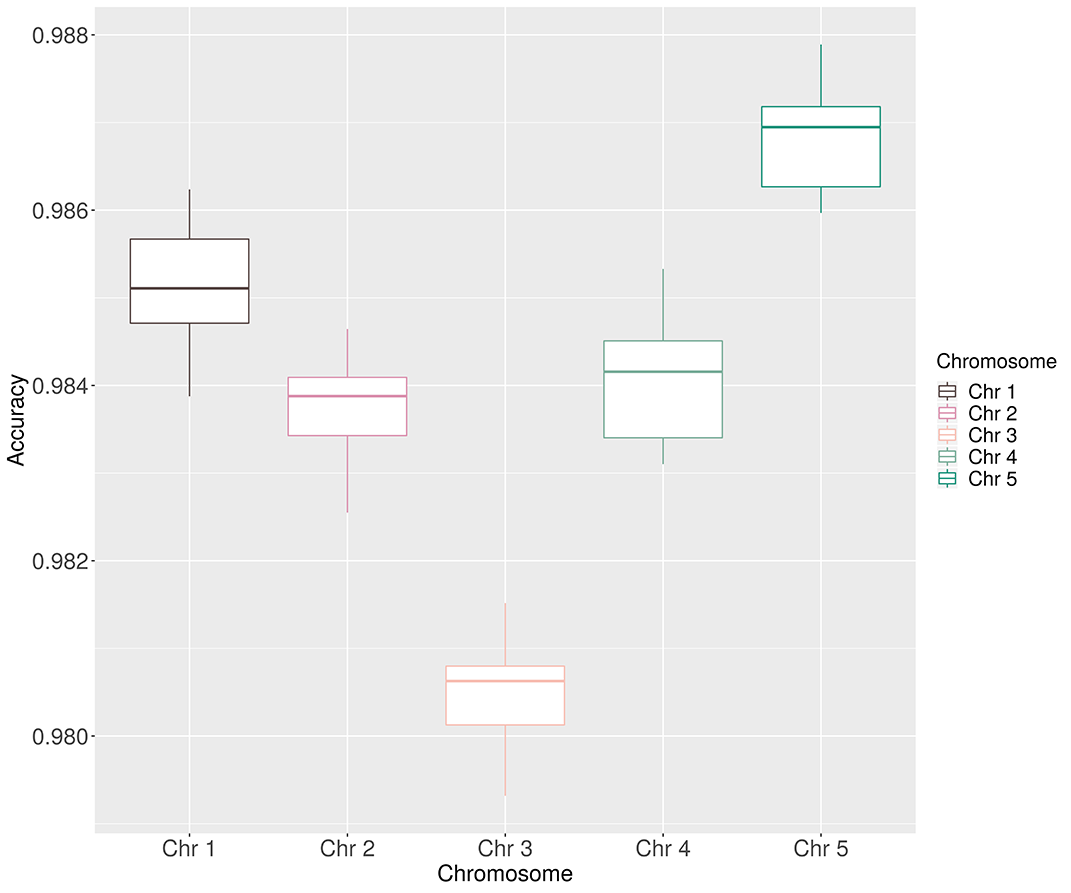

Supplement: Supplementary file 12 — Figure S12. Boxplot showing imputation accuracy for each chromosome across the 20 test sets. [file TPJ-102-872-s012.png]

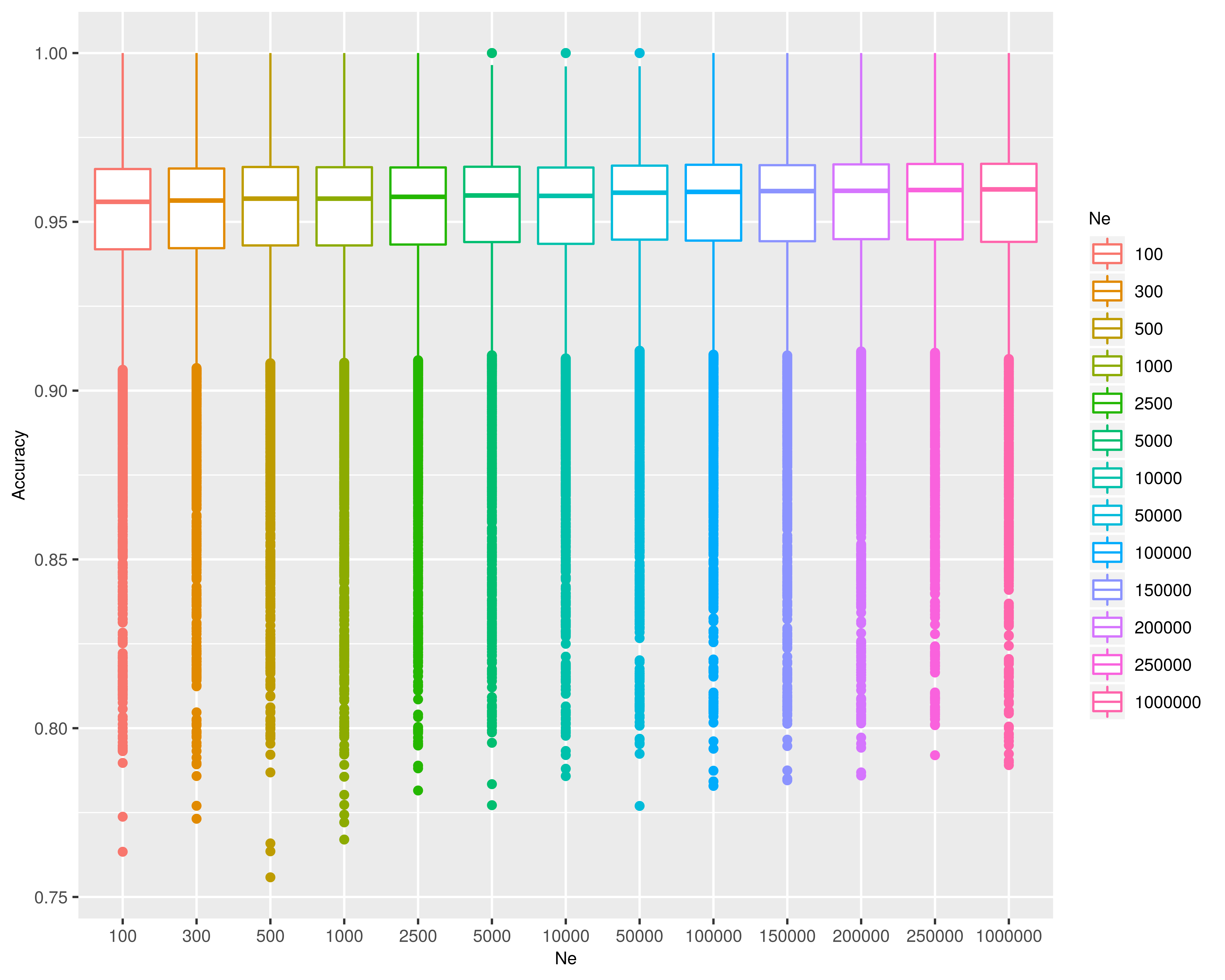

Supplement: Supplementary file 13 — Figure S13. Boxplot displays the imputation accuracy using different effective population size (Ne). [file TPJ-102-872-s013.png]

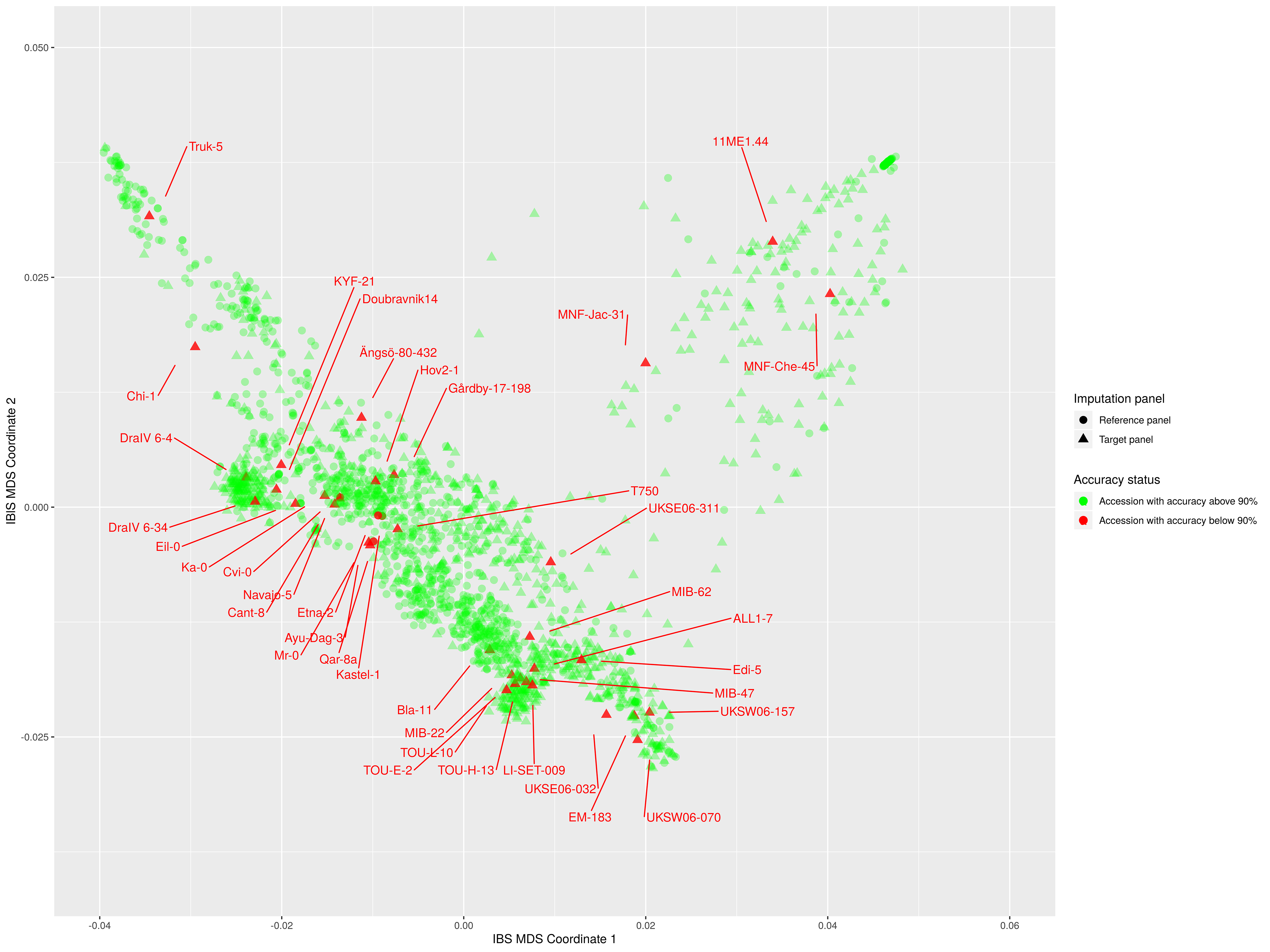

Supplement: Supplementary file 14 — Figure S14. Multidimensional scaling plot illustrating the imputation accuracy and the genetic distances between the 2029 accessions (894 imputed accessions from RegMap and 1135 reference accessions from 1001G). The latter is based on pairwise identity‐by‐state (IBS) distance computed by PLINK 1.9 (Purcell et al., 2007). [file TPJ-102-872-s014.png]
